# Supplementary material for: RNA2Immune: A Database of Experimentally Supported Data Linking Non-coding RNA Regulation to The Immune System
Source: Genomics Proteomics Bioinformatics. 2022 May 17;21(2):283–91. doi: 10.1016/j.gpb.2022.05.001 (PMC10626051; doi:10.1016/j.gpb.2022.05.001)
Supplement: Supplementary Table S1 [file mmc1.docx]

**Table S1 Statistics for the immune function–ncRNA associations in different host species in the RNA2Immune database**

| **Species** | **miRNA** | **lncRNA** | **circRNA** | **siRNA** | **snoRNA** | **piRNA** | **vault RNA** | **atRNA** | **Total** |
| --- | --- | --- | --- | --- | --- | --- | --- | --- | --- |
| *Homo sapiens* | 2742 | 269 | 25 | 6 | 20 | 5 | 3 | - | 3070 |
| *Mus musculus* | 3087 | 272 | 20 | 6 | 4 | - | - | 1 | 3390 |
| *Danio rerio* | 27 | - | - | - | - | - | - | - | 27 |
| *Drosophila melanogaster* | 4 | - | - | - | - | - | - | - | 4 |
| *Gallus gallus* | 22 | 6 | - | - | - | - | - | - | 28 |
| *Rattus norvegicus* | 69 | 3 | - | - | - | - | - | - | 72 |
| *Sus scrofa* | 30 | 12 | - | - | - | - | - | - | 42 |
| *Bos taurus* | 9 | - | - | - | - | - | - | - | 9 |
| *Miichthys miiuy* | 11 | - | - | - | - | - | - | - | 11 |
| *Gadus morhua* | 8 | - | - | - | - | - | - | - | 8 |
| *Macaca nemestrina* | 19 | - | - | - | - | - | - | - | 19 |
| *Marsupenaeus japonicus* | 1 | - | - | - | - | - | - | - | 1 |
| *Canis lupus familiaris* | 1 | - | - | - | - | - | - | - | 1 |
| *Cyprinus carpio* | - | 8 | - | - | - | - | - | - | 8 |
| Total | 6030 | 570 | 45 | 12 | 24 | 5 | 3 | 1 | 6690 |

*Note*: miRNA, microRNAs; lncRNA, long non-coding RNAs; circRNA, circular RNAs; siRNA, small interfering RNA; snoRNA, small nucleolar RNAs; piRNA, piwi-interacting RNA; atRNA, antisense RNA.
