# Supplementary material for: RNA2Immune: A Database of Experimentally Supported Data Linking Non-coding RNA Regulation to The Immune System
Source: Genomics Proteomics Bioinformatics. 2022 May 17;21(2):283–91. doi: 10.1016/j.gpb.2022.05.001 (PMC10626051; doi:10.1016/j.gpb.2022.05.001)
Supplement: Supplementary Table S2 [file mmc2.docx]

**Table S2 Statistics for the immune disease–ncRNA associations in different host species in the RNA2Immune database**

| **Species** | **miRNA** | **lncRNA** | **circRNA** | **siRNA** | **snRNA** | **snoRNA** | **piRNA** | **rRNA** | **tRNA** | **Y RNA** | **vault RNA** | **SRP RNA** | **Total** |
| --- | --- | --- | --- | --- | --- | --- | --- | --- | --- | --- | --- | --- | --- |
| *Homo sapiens* | 20,315 | 4127 | 1029 | 73 | 13 | 123 | 19 | 1 | 21 | 1 | 1 | 3 | 25,726 |
| *Mus musculus* | 5883 | 1021 | - | 50 | - | 3 | - | - | - | - | - | - | 6957 |
| *Sus scrofa* | 1064 | 110 | 131 | 6 | - | 2 | - | 1 | 10 | - | - | - | 1324 |
| *Gallus gallus* | 1068 | 102 | 87 | 4 | - | - | - | - | - | - | - | - | 1261 |
| *Rattus norvegicus* | 688 | 144 | - | 7 | - | 1 | - | - | - | - | - | - | 840 |
| *Bos taurus* | 671 | - | - | - | - | - | - | - | 2 | - | - | - | 673 |
| *Macaca mulatta* | 269 | - | - | - | - | - | - | 2 | - | - | - | - | 271 |
| *Aedes aegypti* | 186 | 6 | - | 6 | - | - | - | - | 1 | - | - | - | 199 |
| *Aedes albopictus* | 134 | - | - | - | - | - | 25 | - | - | - | - | - | 159 |
| *Anopheles sinensis* | 148 | - | - | - | - | - | - | - | - | - | - | - | 148 |
| *Felis catus* | 143 | - | - | 5 | - | - | - | - | - | - | - | - | 148 |
| *Cyprinus carpio* | 120 | - | - | - | - | - | - | - | - | - | - | - | 120 |
| *other* | 753 | 67 | 20 | 2 | - | - | 4 | - | - | - | - | - | 846 |
| Total | 31,442 | 5577 | 1267 | 153 | 13 | 129 | 48 | 4 | 34 | 1 | 1 | 3 | 38,672 |

*Note*: snRNA, small nuclear RNA; SRP RNA, signal recognition particle RNA.
