# Supplementary material for: RNA2Immune: A Database of Experimentally Supported Data Linking Non-coding RNA Regulation to The Immune System
Source: Genomics Proteomics Bioinformatics. 2022 May 17;21(2):283–91. doi: 10.1016/j.gpb.2022.05.001 (PMC10626051; doi:10.1016/j.gpb.2022.05.001)
Supplement: Supplementary Table S3 [file mmc3.docx]

**Table S3 Statistics for the cancer immunology–ncRNA associations in different host species in the RNA2Immune database**

| **Species** | **miRNA** | **lncRNA** | **circRNA** | **Total** |
| --- | --- | --- | --- | --- |
| *Homo sapiens* | 3697 | 583 | 105 | 4385 |
| *Mus musculus* | 369 | 45 | 11 | 425 |
| *Rattus norvegicus* | 20 | 1 | - | 21 |
| *Canis lupus familiaris* | 2 | - | - | 2 |
| Total | 4088 | 629 | 116 | 4833 |
