# Supplementary material for: RNA2Immune: A Database of Experimentally Supported Data Linking Non-coding RNA Regulation to The Immune System
Source: Genomics Proteomics Bioinformatics. 2022 May 17;21(2):283–91. doi: 10.1016/j.gpb.2022.05.001 (PMC10626051; doi:10.1016/j.gpb.2022.05.001)
Supplement: Supplementary Table S4 [file mmc4.docx]

**Table S4 Statistics for the vaccine–ncRNA associations in different host species in the RNA2Immune database**

| **Species** | **miRNA** | **lncRNA** | **Total** |
| --- | --- | --- | --- |
| *Homo sapiens* | 113 | - | 113 |
| *Mus musculus* | 72 | 1 | 73 |
| *Gallus gallus* | 21 | - | 21 |
| *Sus scrofa* | 21 | - | 21 |
| *Ovis aries* | 3 | - | 3 |
| *Bos taurus* | 2 | - | 2 |
| *Macaca mulatta* | 2 | - | 2 |
| *Oncorhynchus mykiss* | 2 | - | 2 |
| *Paralichthys olivaceus* | 1 | - | 1 |
| Total | 237 | 1 | 238 |
|  | | | |
